# The Gendered Effects of Divorce on Mothers’ and Fathers’ Time with Children and Children’s Developmental Activities: A Longitudinal Study

**DOI:** 10.1007/s10680-022-09643-2

**Published:** 2022-11-10

**Authors:** Tomás Cano, Pablo Gracia

**Affiliations:** 1 Universidad Nacional de Educación a Distancia (UNED), Madrid, Spain; 2 Trinity College Dublin, Dublin, Ireland

**Keywords:** Union dissolution, Time use, Life course, Gender inequality, Parenting, Child development

## Abstract

How divorce influences parents’ and children’s time use has received very little scientific attention. This study uses high-quality longitudinal time-diary data across six waves from the *Longitudinal Study of Australian Children* to examine how parental separation shapes parent–child time and children’s daily activities. Results show that separation leads to a strong increase of gender inequalities in parents’ time use. After separation, mother–child time doubles, two-parent time declines by three, and father–child time remains low. Parental separation also leads to a decline in children’s time allocated to educational activities (e.g., studying, reading) and an increase in children’s time in unstructured activities (e.g., TV watching, video gaming, smartphone use). Additionally, the effect of separation on children’s time use is twice as large for boys than for girls, with gender gaps in children’s unstructured time increasing over time. Finally, mother–child time returns to similar pre-separation levels over time, but only after 4 years since separation occurred. The study findings are robust to different panel regression strategies. Overall, this study implies that parental divorce negatively affects children’s developmental time use, especially among boys, and leads lone mothers to experience increasing ‘time penalties’ associated with gender inequalities in society.

## Introduction

The second half of the 20th century saw a dramatic rise in divorce rates, leading to concerns on how separation can impact parent and child well-being (OECD, 2018).^1^ Previous research has found that separation, on average, has negative consequences for mothers’ career progression, paternal involvement, and child development (Kreyenfeld & Trappe, 2020; McLanahan et al., 2013; Lersch & Baxter, 2020). Yet, the explanatory mechanisms linking divorce to well-being are insufficiently understood. Changes in time use and monetary resources have been suggested as two central explanatory mechanisms (Amato, 1993; Carlson, 2006). The present study contributes to a better understanding of one of these two mechanisms by analyzing how parental separation influences parents’ and children’s time use.

Examining how union dissolution relates to changes in time use is important, at least, for two reasons. First, time investments play an essential role in child development (Cano et al., 2019; Hsin, 2009). Thus, if divorce can modify children’s time use and their time with parents, by examining changes in time use after separation we can contribute to a better understanding of inequalities in well-being between children growing up in two-parent families and those raised in one-parent families. Second, divorce has been found to reduce mothers’ income and career progression (Brady et al., 2017; Mortelmans, 2020), largely due to higher maternal caregiving obligations after separation (Kreyenfeld & Trappe, 2020). Therefore, by analyzing how parent–child time changes with separation we can better understand gender inequalities in wages, career trajectories, and well-being in society.

Several studies have analyzed how parental time use differs by family structure. Some studies found that lone mothers spend more time in childcare than partnered parents (Pepin et al., 2018), while other studies did not find sizeable differences in childcare time between single and partnered mothers (Craig & Mullan, 2012). Looking at time use from the child’s perspective, Kalil et al. (2014) found that children in single-mother families receive less total parental time than children in two-parent households. However, this literature has two limitations. First, previous studies were mostly based on cross-sectional data and therefore were unable to control for omitted variable bias. As Pepin et al., (2018, p. 128) concluded, “an ideal dataset would be longitudinal and would capture time in activities”. Second, studies in this field have focused on “what parents do” (Craig & Mullan, 2012; Pepin et al., 2018) or “what children get” (Carlson & Berger, 2013; Kalil et al., 2014), but not on “what children do”. Our study tackles these two important gaps.

The present study on how separation influences parent–child time and children’s daily activities makes three main contributions to the literature. First, we use high-quality longitudinal time-diary data collected bi-annually from parents and children during a 10-year period, adding the most precise existing information on parents’ and children’s time use. Our approach differs from the one adopted in previous studies based on cross-sectional time-use data (Craig & Mullan, 2012), longitudinal data with stylized, coarse measures of parent–child contact (Cheadle et al., 2010), and time-use analyses using few waves of study (Fallesen & Gähler, 2020). Using six waves of cohort panel data allows us to longitudinally observe changes in children’s and parents’ time use before and after divorce over the years, while accounting for unobservable factors in our analyses.

Second, we not only examine how separation influences parent–child time, but also how it shapes children’s time use across activity types, including educational activities (e.g., reading, online study, playing musical instruments, homework) and unstructured activities (e.g., social media, watching TV, unstructured outdoors leisure). Focusing on the child’s activities is critical; research shows that, as children grow up, their time across activity types becomes more relevant for their own development than their parents’ time investments (Del Boca et al., 2017).

Third, we analyze whether there is heterogeneity by gender in how separation impacts children’s time use. Recent evidence shows that having grown up in a lone-parent family has more detrimental effects on adults’ socioeconomic outcomes among men than among women (Chetty et al., 2020). Yet, the factors that may lead boys to perform worse than girls within lone-mother families are poorly understood. One plausible explanation is the potential heterogenous effect of separation on time use between boys and girls. For example, boys and girls may change their daily routines differently after separation, showing gendered processes in their leisure or study habits with direct developmental implications. Also, if father–son time is greater than mother–son time, and fathers often leave the home after separation, boys may be losing more total parental time than girls when divorce occurs. Our study helps to answer these key scientific questions by providing a highly precise analysis of how boys and girls use their time before and after union dissolution.

Overall, our study is, to our knowledge, the first long-term longitudinal analysis of how separation influences parents’ and children’s time use. To accomplish this, we analyze six waves of high-quality, time-diary, cohort data from the *Longitudinal Study of Australian Children* (LSAC) survey. In the following sections, we (1) develop hypotheses drawn from different theories across the social sciences, (2) explain the data and methods used to test our hypotheses, and (3) present and discuss our results within the context of previous research.

## Theoretical Framework

To study the effects of divorce on parental and children’s time investments, we consider both quantity (i.e., total time) and activity type (i.e., the content of time). Regarding the quantity of time, previous studies found a positive effect of parents’ total time on child development, including mother–child time (Del Bono et al., 2016), father–child time (Cano et al., 2019) and both-parent time (Fiorini & Keane, 2014). Yet, parent–child time also adds high pressure and demands among parents, especially among mothers, as they spend more time caring for children than fathers do, despite a narrowing gender gap in recent years (Bianchi et al., 2006; Craig et al., 2006). As for activity type, the activities in which children spend time are essential for their well-being. For example, children’s time spent doing homework or reading books is more productive for their academic outcomes than their time in unstructured activities, such as watching TV (Gracia & Garcia-Roman, 2018; Wight et al., 2009).

We examine two components of time: (a) parental time and (b) children’s time. First, *parental time* (i.e., parent–child’s *quantity* of shared time) comprise four categories: father–child time, mother–child time, two-parent time, and time alone (i.e., without anybody else present). In our measures of mother–child and father–child time, no other adult is present. The quantity of time each parent spends alone with the child indicates high parental responsibility and demands: this care work cannot be shared or avoided as the child’s responsibility rests upon one parent. Contrary to research on father–child contact after separation (Cheadle et al., 2010; Kalmijn, 2015), we examine how separation redistributes time between mother–child, father–child, and two-parent time. We also analyze changes in the quantity of time the child spends without parents or other persons (i.e., time alone).

Second, we look at *children’s time* across activity types (irrespective of whether parents are present or absent in these activities) by distinguishing between two categories of time: educational activities and unstructured activities. Children’s educational time includes activities like reading, doing sports or cultural activities, among others. Children’s unstructured time includes activities like playing video games, watching TV, or non-school-related internet browsing. We select these activities because they can shape heterogeneous pathways in the process of boosting (i.e., educational time) or lowering (i.e., unstructured time) child cognitive development (Cano et al., 2019).

### Parental Time Use

Logically, the quantity of time the child spends with two parents simultaneously will suffer a significant decline after separation. Now, the question is whether parents compensate for the decline of two-parent time, how that compensation is redistributed between mother and father, and how compensation evolves over the child’s life course. It could be that the child gains more parental time after separation, just that the time is distributed differently between mother and father. That would be the case if, for example, mother–child and father–child time increases more than two-parent time declines. These questions remain unexplored in the literature.

Previous studies indicate that individuals’ time use is strongly gendered in ways that may influence the gender division of childcare after divorce. ‘Gender-roles’ theories argue that gendered social norms and everyday interactions lead women to be more involved in childcare than men, with women in heterosexual couples being more focused on time-consuming and emotionally absorbing activities than men (Hays, 1998; West & Zimmerman, 1987). Although lone mothers are likely to experience high levels of time constraints and stress (Haux & Platt, 2021; Meier et al., 2016), the strong persistence of gender ideologies that define childcare primarily as women’s work (Flaquer et al., 2020; Glenn et al., 2016) may indicate an increase in mother–child time after separation (e.g., mothers may compensate for the father’s absence by increasing their time with children after divorce).

Couple’s transition from living together to separation might induce changes in parental identities, particularly for fathers. Identity theory, rooted in symbolic interactionism, separates between *statuses* and *roles* (Stryker, 1968). While statuses refer to social positions, roles refer to the behaviors adscripted to each status. This theory distinguishes between salience of each of the possible roles attached to each status, which might be exacerbated or mitigated by the individual depending on a context or situation (Goffman, 1976). Fatherhood is an example of a status with two clear roles attached to it: provider and caregiver. When couples separate, ambiguity over fathers’ roles tends to increase, possibly leading to declines in childcare time allocation (Rane & McBride, 2000). Another important mechanism behind possible changes in father–child time after separation is *time availability* (Coverman, 1985). In Australia, mothers take the child’s custody in nine out of ten separated couples, where fathers move out from home: this imposes physical, temporal, and monetary limits for fathers’ childcare time investments (e.g., commuting costs, finding a new house) (Smyth & Chisholm, 2017). These arguments lead to the expectation that father–child time will decline after separation.

The amount of time children spend alone should not lead to important changes after parental separation. Time alone is an interesting measure by itself for the literature on child and adolescent well-being. Previous studies have suggested that time alone reflects the degree of independence and autonomy that young people achieve in their daily lives, while bringing both opportunities and challenges for their present and future well-being and lifestyles (Gracia et al., 2020). Pasteels and Bastaits (2020) found that children who experienced parental separation do not differ in how they perceive their time alone compared to children in two-parent families. However, there is no existing research, to our knowledge, that has investigated the way children’s time alone changes after parental separation occurs. We expect that, if it is true that mothers compensate for the loss of resources and fathers’ absence by increasing their time with children after separation, one should observe stability in children’s time spent alone after divorce.

Previous research examined post-separation levels of parent–child contact and involvement. Using a growth curve mixture to model trajectories of father–child contact after dissolution in the US, Cheadle et al. (2010) found that about two-fifth of fathers maintained high levels of contact after divorce, while one-fifth of fathers became largely absent. Although father–child contact after divorce has increased over recent years (Westphal et al., 2014) and “new” fathers are increasingly gender egalitarian (Cano, 2019), most studies suggest that father–child time declines after separation (Carlson, 2006; Furstenberg et al., 1983; Koppen et al., 2018; Pardo et al., 2019). Evidence for mothers is mixed. A study using fixed effects models with US data found that partnered mothers are more involved in the child’s schooling activities and shared activities at home, compared to single mothers (Ressler et al., 2016). By contrast, a longitudinal study using German data found that mothers’ involvement remains quite stable after separation (Gratz, 2017). Cross-sectional time-diary studies with accurate time-use data provide inconclusive results too. While an Australia study found that single and partnered mothers spend similar amounts of time with children (Craig & Mullan, 2012), a US study found single mothers to spend more time with children than partnered mothers (Kalil et al., 2014). Our study differs from all these previous studies in that we apply a longitudinal time-diary approach. Drawing on our previous theoretical assumptions, we expect:

#### Hypothesis 1

Parental separation leads to a decline in father–child and two-parent time and an increase in mother–child time, without altering the child’s time spent alone.

### Children’s Time Across Activity Types

Several theoretical explanations indicate that parental separation might modify children’s time across activity types. The ‘parental resource perspective’ (Coleman, 1988) and the ‘family investment model’ (Astone & Mclanahan, 1991) suggest that separation can harm family resources by reducing money (e.g., relocation costs, new expenses) and time (e.g., commuting costs for the absent parent, new job demands, new family obligations) (Amato, 1993; Carlson, 2006). Declines in parental socio-economic resources should also reduce the amount of time parents can invest in managing or supervising children’s educational activities (e.g., homework, private lessons), as opposed to unstructured activities (e.g., screen-based time), which are economically less costly and likely to increase as parents become less present at home. Therefore, separation might discourage children’s time in educational activities, as these activities crucially depend on parents’ time availability, persistence, and resources to invest in children (Gracia and Garcia-Roman, 2018; Lareau, 2011).

Parental separation can also increase stress levels within families and this, in turn, may contribute to changes in children’s own activities. The ‘divorce stress-adjustment perspective’ (Amato, 1993) suggests that during the process of separation, parents and children experience higher stress levels, a reacting condition linked to lower levels of psychological well-being, life satisfaction, and increase in conflict. A significant group of parents and children experience higher levels of stress during the process of marital breakup (Booth & Amato, 1991; Harkonen, 2017). For parents, stress can lower energy, capacity, and self-efficacy to supervise children and to engage in positive parenting (Haux & Platt, 2021; Kiernan & Huerta, 2008). For children, lower levels of psychological well-being or experiencing more family conflicts at home might induce to higher temptation of participating in unstructured leisure activities, like watching TV or engaging in social media. Overall, we expect:

#### Hypothesis 2

Parental separation leads to an increase in the child’s time in unstructured activities, and a reduction in the child’s time in educational activities.

### Heterogeneous Effects by Child Gender

Previous research suggests that the child’s gender can moderate the effect of parental separation on parents’ and children’s time investments. First, parents’ time investments differ by the child’s gender. Across countries, mothers have been found to spend more time with daughters, and fathers with sons, reflecting gendered socialization processes, as well as higher internal satisfaction levels, peer pressure, social expectations, and mechanisms of comparative advantage (Bonke & Esping-Andersen, 2011; Harris & Morgan, 1991; Lundberg, 2005; Raley & Bianchi, 2006). Following this logic, if most single-parent families are headed by the mother, an increase in mother–child time after separation may favor mother–daughter time, while father–son time would show a greater decline than father–daughter time. While previous research found that fathers remain more involved with sons than with daughters after separation (Bastaits et al., 2015; Gratz, 2017), these studies did not apply a time-use approach that allows to accurately observe changes in mother–child and father–child time. Overall, we expect:

#### Hypothesis 3a

The increase of mother–child time after separation is greater for girls than for boys, and the decline in father–child time is greater for boys than for girls.

Second, children’s time investments are also gendered. Time-use research shows that boys spend more time in unstructured activities like screen-based leisure time, while girls spend more time in educational activities (Bohnert and Gracia, 2021; Gracia et al., 2022; Wight et al., 2009). Parental separation might amplify these gender differences in children’s time use. While studies have often found small gender differences in how parental separation affects child development (Amato, 2010), paternal absence was found to induce boys to higher aggressiveness or anti-school attitudes, with boys showing greater difficulties than girls in adjusting to divorce in the new family arrangements, often in single-mother families (Hetherington & Stanley-Hagan, 1997; Legewie & DiPrete, 2012; Rutter, 1987). Ethnographic research reveals how adolescent girls under specific processes of family change embrace a feminine identity based upon studying hard, being attentive and caring for others (Epstein, 1998). By contrast, boys might demonstrate a masculine identity in changing social or family processes by “taking pride in their lack of academic effort” (Morris, 2008, p. 736). These exaltations of gender-role behaviors are likely to be strengthened after parental separation. Therefore, we expect:

#### Hypothesis 3b

After separation, boys show a sharper increase in time spent in unstructured activities and a greater decline in time spent in educational activities than girls.

### A Life-Course Approach

Does the effect of separation on parents’ and children’s time investments increase, decrease, or persist over the years? This question remains unanswered. Previous studies on the effect of separation on income and on mental health show strong negative effects in the short run, but with a recovery to pre-separation levels after around one year since separation (Booth & Amato, 1991; Leopold, 2018; Leopold & Kalmijn, 2016). Such timing of levelling up income and stress to pre-separation levels might be paralleled in terms of time investments. According to *set-point theory*, while most individuals have a quite stable baseline in their lifestyles, key life events can modify their behaviors in the short term, before reverting to their baseline (or set-point) through adapting to new circumstances over the years (Brickman & Campbell, 1971; Diener et al., 2009).

As a result, two-parent time may recover after the years since separation. It could be that former partners reduce their conflict with the years, meaning that they may start to share some moments of the week with children. Also, after several years since separation, parents may be more likely to start new partnerships who may become social parents that are involved in childcare (Kalmijn et al., 2019). Consequently, maternal time could steadily decline to get closer to pre-separation levels, in parallel with a recovery of two-parent time over the years since separation. Consequently, we expect:

#### Hypothesis 4a

After the years since separation, there is a steady increase in two-parent time and a steady decline in mother–child time, getting closer to pre-separation levels.

Previous studies have not analyzed how parental separation influences children’s daily activities by considering both the short and longer term. The most important shocks in resources, family stress and daily routines after separation happen in the short run (e.g., Booth & Amato, 1991). After such initial shock, parents and children tend to stabilize their material uncertainty and emotional concerns, instead of getting locked in a persistent negative state. Therefore, we expect:

#### Hypothesis 4b

The effect of parental separation on children’s educational and unstructured activities is strong when separation occurs (short-run), but it stabilizes in the subsequent years (long-term).

## Method

### Data and Sample

We use data from the *Longitudinal Study of Australian Children* (LSAC), a biennial survey that started in 2004 including two cohorts of approximately 5000 Australian children each, one born in 2000 (“Kindergarten Cohort”) and another one born in 2004 (“Birth Cohort”) (Australian Institute of Family Studies 2002). The LSAC is internationally unique in that it offers rich longitudinal time-diary data for each interview year. Time-diary data cover individuals’ daily activities on a 24-hour framework, providing more reliable and less biased data than stylized questions asking “how often” respondents engage in activities (Bianchi et al., 2006; Fisher et al., 2012; Kan, 2008).

We restricted our analyses to six waves of the ‘Kindergarten Cohort’, which covers information from age 4 (2004) to age 14 (2014). Time diaries in the LSAC were designed to change over time and are therefore adapted to children’s developmental processes. In waves 1–3 (ages 4–8), as children were very young, parents filled out two “light diaries” (Hofferth et al., 1997). These light diaries split the day into 15-minute time intervals, with a total of 96 time slots. In waves 4–6 (ages 10–14), time diaries were no longer filled out by parents, and instead children filled the diaries themselves. In waves 4–6, children wrote down what they were doing during the 24 hours in a structured temporal sequence. The day after the interview, interviewers coded information provided by children into a pre-defined list of activities and collected information about who was the child with and where.^2^ The ‘Kindergarten Cohort’ contains time-diary information on ‘with who’ children spent time across all six waves (e.g., alone, with father, with mother or both parents) (Corey et al., 2014; Mullan, 2014).

We restrict our main analysis to weekdays. Waves 1–3 included two time-diaries per child (one for weekdays and one for weekends), but waves 4–6 only included one diary per child, where weekends were significantly underrepresented (Mullan, 2014,  p. 15). This may hamper comparability across waves. Therefore, by focusing only on weekdays, our analyses reduce this source of bias across waves, as the number of weekday’s diaries is stable across all waves. While weekend diaries are relevant too, studying children’s daily routines during weekdays is crucial, as it is during weekdays when many children engage in regular sets of routines that are essential for their subsequent development (Fiorini & Keane, 2014). Although our main analyses concentrate exclusively on weekdays for data-related reasons, we also provide some additional analyses focusing on weekends for waves 1–3. Most time-diaries were collected between mid-March and the end of September to skip summer school holidays in Australia (December–January).

Our sample included all diaries collected by children who in wave 1 (age 4) were living with two biological different-sex parents. We excluded diary observations with missing information in living arrangements (*n* = 3 diaries), mother’s or father’s level of education (*n* = 413 diaries), transitioning to a single-parent house due to parental death (*n* = 67 diaries), those who entered the survey already with separated parents or living in other arrangements than two biological parents (*n* = 2713 diaries), and those who mistakenly had duplicate diaries (*n* = 35 diaries). After these exclusions, we ended up with a total of 14,862 observations from 3719 children.

Analyses included two subsamples. The *separation sample* contains all children who experienced a transition from living with two parents to living with just one parent (*n* = 2054 observations from 505 children). The *partnered sample* includes all children observed in two-parent homes across the six waves (*n* = 12,808 observations from 3214 children). Keeping the ‘partnered sample’ in our analyses has two main advantages: (a) experiencing divorce is a selective life-course transition (i.e., the lower-educated separate more than the higher-educated) and so keeping both samples allows us to observe selective differences between families who experience separation and those who do not; (b) by keeping the partnered sample we increase the final sample size, allowing for better effect’s estimations in general, and for an age effect estimation in particular (Leopold, 2018).

### Dependent Variables: Measures of Time Use

The LSAC allows us to examine both the *quantity* (e.g., parent–child total daily minutes) and *content* (e.g., reading/studying) of time. Our dependent variables are constructed by following a comparable harmonization across the six waves of study, drawing on previous studies and reports (see Table 3) (Cano et al., 2019; Corey et al., 2014; Mullan, 2014). We use a total of six dependent variables. Four dependent variables capture parental time investments: (1) *Two-parent time*: child was with mother *and* father together; (2) *Mother–child time*: child was with mother and without father present; (3) *Father–child time*: child was with father and without mother present; (4) *Time alone*: child was without any parent or other person present.^3^ The other two dependent variables capture children’s time investments (irrespective of whether parents are present or absent in these activities), which measure child total daily minutes allocated to two groups of relevant activities: (5) *Educational time* includes cognitively stimulating activities performed outside school (e.g., reading, studying, doing puzzles, playing music, going to libraries); and (6) *Unstructured leisure time* includes non-structured leisure activities (e.g., watching TV, non-structured play, mobile phone texting).^4^,^5^

### Independent Variable: Parental Separation

Our independent variable is *parental separation*. We identify parental separation by a change in the child’s household composition from “living with two biological parents” to *not* “living with two biological parents” (in 95% of the cases the first transition is from living with two different-sex biological parents to living only with the biological mother, and 5% transit to living only with the biological father).

To capture temporal dynamics of parental separation on time investments (i.e., short- and long-term effects), we split our independent variables in two: (a) a *dummy* variable changing from 0 (observations without separation) to 1 (with separation), and (b) a *categorical* variable identifying time-use before and after separation, where 0 is the first observation after separation. To prevent cells with low number of cases, we recode the duration categorical variable into five categories of time before and after separation: 10–8; 6–4; and 2 years *before* separation; the first observed year after separation (0); 2; and 4 or more years *after* separation. As in Leopold (2018), observations of the ‘partnered sample’ are set in the reference category (i.e., 10–8 years before divorce/separation).

### Control and Moderator Variables

We consider multiple covariates that are potential confounders in the effect of parental separation on time investments: *State* (categorical), *Siblings at home* (dummy); *Language spoken at home* (English or not: dummy); *Re-partnering* (whether a non-biological parent entered the home after separation); *Father’s/mother’s education* (University degree or not); *Child age*, as a categorical variable to avoid collinearity with duration before or after separation (ages: 4, 6, 8, 10, 12 and 14); *Father’s/mother’s SES*, based on four categories recoded from the Australian and New Zealand Standard Classification of Occupations (ANZSCO) (Australian Bureau of Statistics, 2013): (1) Managers and professionals; (2) Intermediate class (i.e., skilled technicians, non-manual works in sales, commerce and administration); (3) Working class (i.e., production workers, routine workers in industry or service); No occupation (i.e., unemployed or inactive). Finally, *child’s sex* (girl/boy at birth) is our moderator variable to investigate gender differences in the effects of separation on time investments.

Table 1 shows the study measures’ means and standard deviations for the whole sample, the partnered sample and the separated sample. Besides providing descriptive evidence on the dependent variables by family structure, Table 1 shows that children in separated families are overrepresented in socioeconomically disadvantaged households, especially when looking at fathers’ characteristics. These distributions indicate the relevance of controlling for unobservable factors driving both the propensity of family instability and the child’s time across activity types.

**Table 1:** Summary statistics

	Partnered sample		Separated sample		Diff.
	Mean	*SD*	Mean	*SD*	*p*
Dependent variables (in daily minutes)					
Mother–child solo time	164.32	194.74	212.98	240.38	***
Father–child solo time	39.97	85.89	41.84	109.82	
Two-parents’ time	157.51	231.95	124.94	231.97	***
Time alone	236.42	253.24	222.31	252.93	*
Educational activities	122.48	103.34	115.57	108.31	*
Unstructured leisure	139.07	119.54	150.06	121.70	***
Independent and control variables					
Father has a college degree	0.35		0.24		***
Mother has a college degree	0.39		0.27		***
Father is not employed	0.04		0.06		
Father has a working-class occupation	0.40		0.55		***
Father has an intermediate occupation	0.17		0.14		**
Father has a managerial/professional job	0.39		0.25		***
Mother is not employed	0.24		0.26		*
Mother has a working-class occupation	0.31		0.31		
Mother has an intermediate occupation	0.18		0.19		
Mother has a managerial/professional job	0.27	0.44	0.22	0.42	***
Child’s age in months	114.99	41.36	113.56	41.29	
Child is a girl	0.49		0.52		*
Child speaks English at home	0.90		0.94		***
At least one other child in the household	0.45		0.44		
State of residence:					
New South Wales	0.31		0.30		*
Victoria	0.25		0.23		
Queensland	0.20		0.23		**
South Australia	0.07		0.07		
Western Australia	0.11		0.08		**
Tasmania	0.03		0.03		
Northern Territory	0.01		0.02		
Australian Capital Territory	0.03		0.03		
Re-partnering	0.00		0.09		***
Number of observations	12808	2054			
Number of children	3214	505			

Longitudinal study of Australian Children. *K* Cohort, waves 1–6 (2004–2014)

Observations are pooled across waves. Difference column refers to differences between partnered and control separated samples, using *t* test. Levels of significance: *p < 0.05, **p < 0.01, ***p < 0.001

### Empirical Strategy

We start with the following linear model for repeated observations nested within children:

1 $$f\left( {T_{{{\text{it}}}} } \right) = \alpha + \beta_{1} {\mathbf{FB}}_{{{\text{it}}}} + \beta_{2} {\varvec{X}}_{{{\text{it}}}} + \beta_{3} {\varvec{G}}_{i} + \eta_{i} + e_{{{\text{it}}}}$$

where *T* is time (alone, with father, mother, both parents, and across activity types) of child *i* in year *t,* and *α* is the intercept. **FB** contains a dummy and a continuous indicator of parental union dissolution. *β*_*1*_ indexes the coefficients of main interest to test our working hypotheses. ***X*** is a vector of time-changing control variables, while ***G*** is child’s sex, our only time-constant variable. *β*_*2*_ represents the coefficients of control variables and *β*_*3*_ represents the coefficients of child’s sex; *η* represents person specific time-constant unobserved factors affecting selection into divorce and time use; *e* is the random disturbance across *i* and *t*.

First, we use random effects (RE) generalized least squared regressions (GLS). Random effects models are especially suitable for our research because they allow us to obtain main effects of time-invariant variables, and our moderator variable is time-invariant (i.e., child’s sex). This is particularly relevant, as pre-divorce time-use of boys and girls may differ (Mencarini et al., 2019). However, random effects GLS models impose the strong assumption that *e*_it_ and *η*_i_ are not correlated with divorce and time investments (Allison, 1994). Also, the probability of divorce could be associated with time-use through time-constant unobservable factors, leading to selection bias.

We further estimate fixed effects models (FE). FE models are more robust than RE models and they partially avoid self-selection bias of time-constant unobservable factors (Wooldridge, 2010). The FE model takes the form of:

2 $$f\left( {T*_{{{\text{it}}}} } \right) = \beta_{1} {\mathbf{FB}}*_{{{\text{it}}}} + \beta_{2} {\varvec{X}}*_{{{\text{it}}}} + e*_{{{\text{it}}}}$$

The robustness of FE models comes at a price: in this equation, ***G*** drops because child gender is a time-invariant variable that cannot be estimated. As noted, *η*, because is time-invariant, also drops from the equation. Therefore, the coefficients of interest (i.e., *β*_*1*_) are not affected by any observed (or unobserved) time-invariant factors correlated with **FB** and ***T***. This eliminates selection bias due to time-invariant unobservable factors. In Eq. (2), T*_it,_ FB*_it_ and X*_it_ are time-variant differences from the individual means. Thus, this model tells us how changes over time in individuals’ characteristics affect changes in time use. That is, individuals act as their own statistical adjustment. Therefore, fixed effect models account for potential bias encountered in the RE models. Amato and Anthony (2014, p. 373) wrote: “Given the impossibility of conducting true experiments, child fixed effects models are one of the best available methods for estimating the causal effect of divorce on children”.

Fixed effect models also have limitations for the type of research that we conduct in this study. FE models control for children’s unobserved characteristics, but they also assume that unobserved characteristics are constant over time. This can be problematic in this study, as we want to analyze the effects of parental separation on time use in a longitudinal fashion, and children and their unobserved characteristics change significantly from childhood to adolescence. In addition, FE models are particularly sensitive to measurement error, which might be another source of bias in our estimates. FE models also have the limitation that they only compare before–after changes of children who experienced parental separation. This leads to excluding from our FE analytical sample those children who do not experience separation. That means not being able of comparing “treated” children with a “control” group of children. Because of these limitations, we also have a third empirical strategy, besides RE and FE, namely value-added models (we explain value-added models below). We are aware that no empirical strategy to control for unobserved heterogeneity that affect both parental separation and time use can ever be completely convincing. Therefore, we opt for using three different empirical strategies, showing all our results, and discussing the advantages and disadvantages of each method.

We estimated multiple models for each of our six dependent variables by firstly estimating the short-term separation average effects (i.e., with the dummy variable of separation) and, secondly, the long-term separation effects (i.e., with the categorical variable of separation duration). As indicated, we test our four hypotheses using both FE and RE models. We finally conduct several sensitivity analyses.

## Findings

### Results on Fathers' and Mothers' Time with Children

**Fig. 1:**
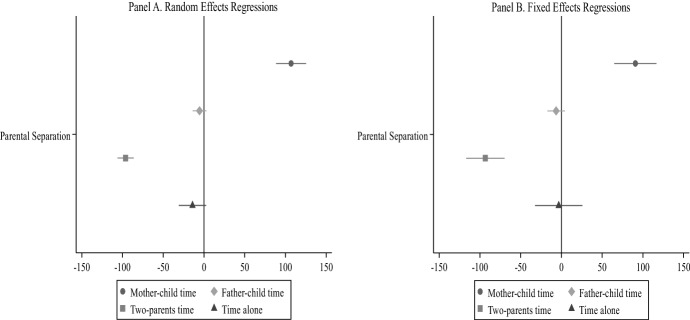
Regression models of children’s time with parents and alone—average effect. *Source* Longitudinal Study of Australian Children. K Cohort, waves 1–6 (2004–2014). *Note* Both the random effects (Panel A) and fixed effects (Panel B) regressions include four separate models, controlling for child age, maternal education, paternal education, mother’s class, father’s class, language spoken at home, number of siblings, region of residence, and residential parent re-partnering. Child’s sex is only included in the random effects models. Confidence intervals are included at the 95% level (number of diary observations = 14,862)

**Fig. 2:**
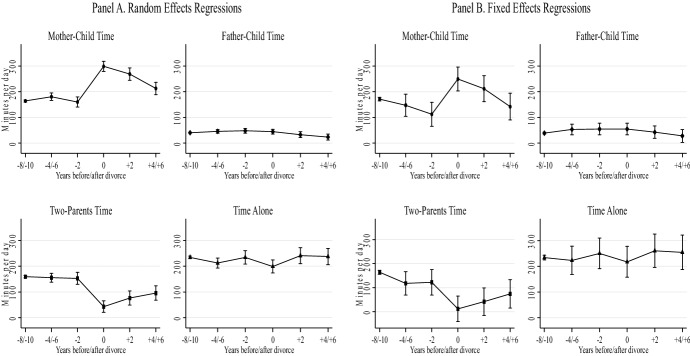
Regression models of children’s time with parents and alone—long-term effect. *Source* Longitudinal Study of Australian Children. *K* Cohort, waves 1–6 (2004–2014). *Note* Both the random effects (Panel A) and fixed effects (Panel B) regressions include four separate models, controlling for child age, maternal education, paternal education, mother’s class, father’s class, language spoken at home, number of siblings, region of residence, and residential parent re-partnering. Child’s sex is only included in the random effects models. Confidence intervals are included at the 95% level (number of diary observations = 14,862)

Figures 1 and 2 present the first set of regression models on the effect of separation on children’s time with mother, father, two parents and alone. Figure 1 shows the *average effect* of separation (presented as regression coefficients). Figure 2 shows the *long-term effect* of separation across the years before and after the event (presented as predicted values). Panel A shows the results for the random effect regressions, while Panel B illustrates the fixed effects regressions. The full models are shown in Tables 4 and 5.

Figure 1 (Panel A) shows that separation is strongly associated with changes in parent–child time. Consistent with Hypothesis 1, we observe a sharp increase in mother–child time after separation, accounting for 107 daily minutes (*p* < 0.001). This occurs at the cost of two-parent time, which shows a sharp decline of 96 daily minutes (*p* < 0.001). By contrast, father–child time remains unchanged after separation, being reduced only by 5 min per day. Time alone experiences a small decline of 14 min. Panels A and B (random and fixed effects, respectively), show similar results.

Figure 2 shows relevant variations in the long-run effects of separation on parent–child time. Panel A (random effects) shows that the strong positive effect of separation on mother–child time is concentrated in the first observation after divorce (i.e., year 0 in the graph), with major increases of nearly 2 hours per day. Mother–child time takes 4 years until reaching almost the same pre-separation levels. By contrast, father–child time barely changes with separation, with a slow, unsubstantial and non-significant decline over the years. Two-parent time decreases dramatically in the immediate separation year, but it increases in the following years. Finally, time alone returns to pre-divorce levels after 2 years since separation, but with minor changes. Again, Panels A and B (fixed and random effects) show similar results.

To contextualize the gendered nature of these results: before separation, mothers spent circa 150 daily minutes on mother–child time on a random weekday, while fathers spent an average of 50 min on father–child time; that is, three times less. These figures are consistent with Craig’s (2006) cross-sectional study with Australian time-diary data. Our analyses show longitudinally that, after separation, the gender gap in parent–child time jumps from three to seven times, moving to an average of 350 min among mothers and 50 min among fathers.

### Results on Children’s Developmental Activities

**Fig. 3:**
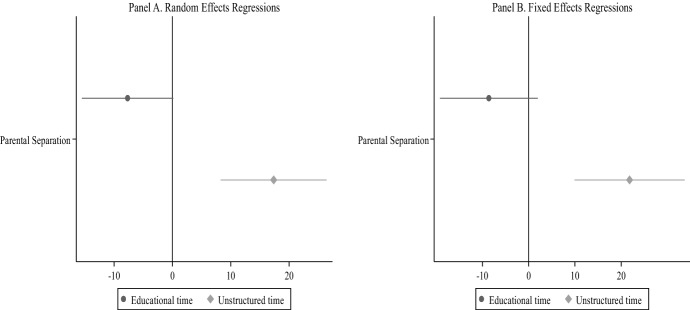
Regression models of Children’s time in educational and unstructured activities—average effect. *Source* Longitudinal Study of Australian Children. K Cohort, waves 1–6 (2004–2014). *Note:* Both the random effects (Panel A) and fixed effects (Panel B) regressions include two separate models, controlling for child age, maternal education, paternal education, mother’s class, father’s class, language spoken at home, number of siblings, region of residence, and residential parent re-partnering. Child’s sex is included in the random effects models only. Confidence intervals are included at the 95% level (number of diary observations = 14,862)

**Fig. 4:**
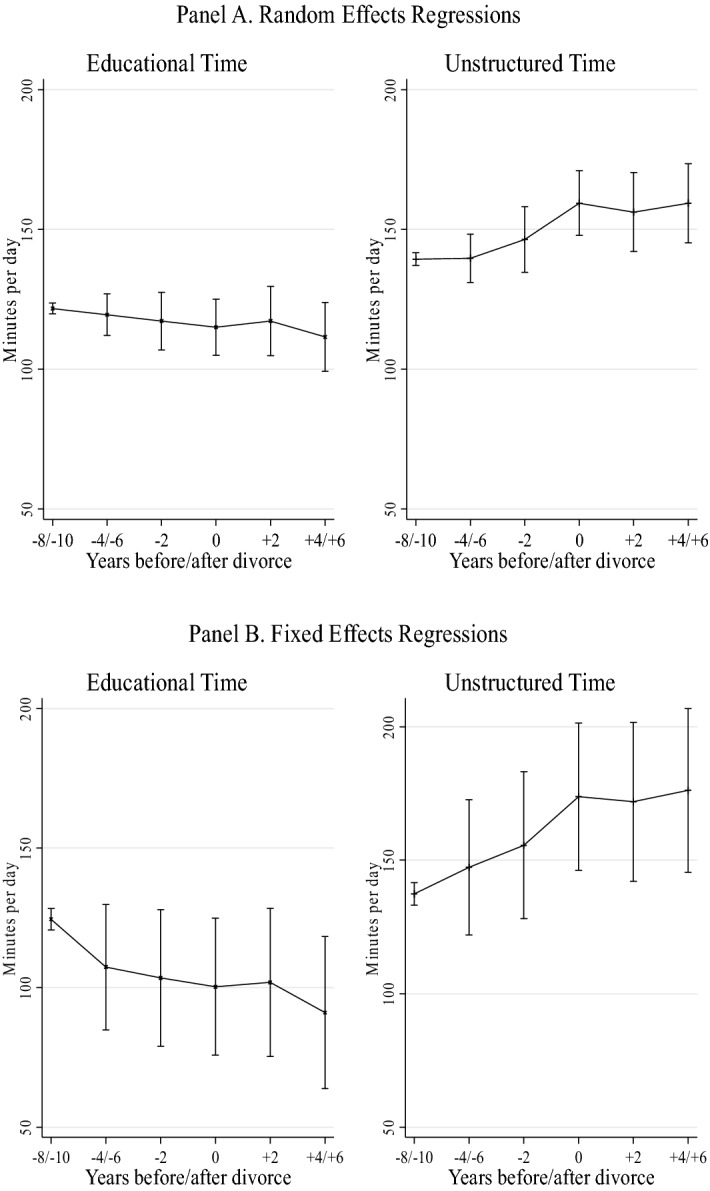
Regression models of children time in educational and unstructured activities—long-term effect. *Source* Longitudinal Study of Australian Children. *K* Cohort, waves 1–6 (2004–2014). *Note:* Both the random effects (Panel A) and fixed effects (Panel B) regressions include two separate models, controlling for child age, maternal education, paternal education, mother’s class, father’s class, language spoken at home, number of siblings, region of residence, and residential parent re-partnering. Child age is included in the random effects models only. Confidence intervals are included at the 95% level (number of diary observations = 14,862)

Figures 3 and 4 show the effects of parental separation on children’s time in educational activities and unstructured leisure. Figure 3 presents the average effects models and Fig. 4 the long-term effects, with the two figures showing both random and fixed effects models. Results are generally in line with Hypothesis 2, particularly for unstructured leisure activities. Figure 3 (Panel A) shows that parental separation leads children to increase their unstructured leisure time by 17 min a day (*p* < 0.001) and to a smaller decrease of 8 daily minutes in educational activities. The results of random and fixed effects models (Panels A and B, respectively) show again similar results. In Fig. 4, we observe a negative trend in children’s time on educational activities after parental separation and a positive trend on unstructured leisure activities, but such a trend stabilizes 2 years after divorce.

### Results on Heterogeneous Effects by Child Gender

**Fig. 5:**
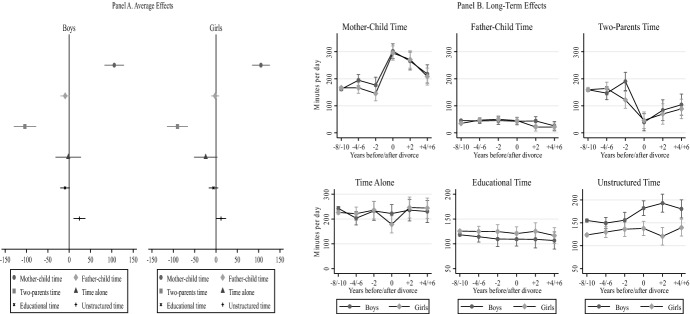
Random effects regression models of child time use by child gender. *Source* Longitudinal Study of Australian Children. K Cohort, waves 1–6 (2004–2014). *Note* Panel A shows regression estimated separately in two subsamples of girls and boys (i.e., 12 regressions, six per girls and six per boys). Panel B shows six regressions where each of them includes an interaction term between child’s sex and separation in a categorical form that indexes years before and after. All models control for child age, maternal education, paternal education, mother’s class, father’s class, language spoken at home, number of siblings, region of residence, and residential parent re-partnering. Confidence intervals are included at the 95% level (number of observations = 14,862)

Figure 5 illustrates the random effects models to test Hypothesis 3. Panel A shows average effects of separation for all study six dependent variables estimated separately for the subsample of boys and the subsample of girls. Panel B presents the long-term effects of separation on the same six dependent variables, interacting the variable ‘child sex’ with ‘time since divorce’. For reasons of space, in this section we only report results from random effects. Results from fixed effects models were consistent with those from random effects.

Figure 5 shows results that support Hypothesis 3 in various respects. In Panel A (average separation effects) of Fig. 5, we observe that boys, compared to girls, lose more father–child solo time (*β* = − 9 vs − 2 daily minutes) and more two-parent time (*β* = − 104 versus − 91 daily minutes). Also, boys’ time alone remains stable after divorce, while time alone for girls declines by 24 daily minutes. Regarding activity types, after parental separation, boys increase their time in unstructured leisure activities by 31 daily minutes, while girls increase it by 14 min. We see that boys reduce their time in educational activities by 10 daily minutes after separation, with girls reducing their educational time by 5 min only. Interestingly, unlike predictions, mother–child solo time increases equally for boys and girls after divorce.

In Panel B (long-term effects) of Fig. 5 we observe that only boys increase two-parent time before divorce, but gender differences in two-parent time disappear afterward. Two years after separation, boys keep the same levels of father–child solo time and girls drop their father–child solo time, but gender differences in father–child solo time vanish after 4 years since separation. Gender gaps in unstructured leisure time increase remarkably over the years since separation. Before divorce, boys spend 156 daily minutes in unstructured leisure, with girls spending 136 min in the same activities (14% gender gap). Two years after separation, boys spend 182 daily minutes in unstructured leisure and girls 137 (28% gender gap). After 4 years since divorce, boys spend 193 daily minutes in unstructured leisure, while girls only 121 min (46% gender gap).

### Additional Analyses I: Separation Effects across Children’s Ages

We additionally tested if the effect of parental separation on time use differs across age groups. The key reason for estimating these additional analyses is that fixed and random effects models assume child unobserved heterogeneity to be time constant across the 10 years of observation (i.e., from age 4 to 14). But, as noted, children’s unobserved characteristics may significantly change during the observed period, potentially affecting our estimates of the effect of separation on time use. Therefore, analyzing separation’s effects on time use across child age groups helps solving this issue by allowing age-specific unobserved heterogeneity.

We split the main sample into five subsamples of transitions: ages 4–6; ages 6–8; ages 8–10; ages 10–12; ages 12–14. Following Todd and Wolpin (2007), we capture child cumulative processes of learning by calculating the effect of separation on time use in the five subsamples using five separated value-added models. Value-added models control for each of the dependent variables at *t*−1. In this way, current time investments depend on previous time investments and previous parental inputs, thus capturing the cumulative dimension of learning across developmental processes.

Table 2 presents the results of the value-added models.^6^ Results confirm the heterogeneous effects of separation on time investments across child age-specific developmental stages. Estimates of parental time use decrease in magnitude as the child grows up. On the contrary, estimates of children’s time use increase in magnitude as the child grows up. This is coherent with the notion that, as children grow up, they spend less time with parents and more time on their own. Results of this additional analysis do not change the conclusions of this study, but they show a more nuanced picture: the younger the child when separation occurs, the larger the effect of divorce on parent–child time; and the older the child, the larger the effect on children’s time across activity types. That means that the timing of divorce has heterogeneous impacts across different types of time use.

**Table 2:** Value-added regression models of child time-use by child age

	Mother–child solo time		Father–child solo time		Two-parents’ time		Time alone		Educational activities		Unstructured activities	
	*β*	SE	*β*	SE	*β*	SE	*β*	SE	*β*	SE	*β*	SE
*Panel A: age 4–6*												
Separation	225.65***	28.07	67.25***	13.15	− 187.91***	34.74	− 144.81***	39.24	− 10.29	11.46	4.04	8.27
Intercept	189.60***	21.65	40.54***	10.14	228.27***	26.80	160.38***	30.27	114.57***	8.84	71.01***	6.38
Controls	Yes		Yes		Yes		Yes		Yes		Yes	
*R*^2^	0.07		0.03		0.09		0.18		0.05		0.07	
Number of observations	2104		2104		2104		2104		2104		2104	
*Panel B: age 6–8*												
Separation	252.81***	18.31	21.92*	8.40	− 209.01***	23.34	− 99.09***	24.79	− 8.99	7.45	4.67	5.75
Intercept	188.28***	14.23	42.73***	6.33	181.02***	17.84	117.84***	18.23	111.14***	5.9	64.23***	4.75
Controls	Yes		Yes		Yes		Yes		Yes		Yes	
*R*^2^	0.07		0.02		0.10		0.18		0.04		0.07	
Number of observations	4105		4105		4105		4105		4105		4105	
*Panel C: age 8–10*												
Separation	146.49***	11.98	− 4.27	5.09	− 128.11***	14.05	− 40.15**	15.65	− 10.66*	5.77	10.39	7.05
Intercept	214.18***	13.27	45.10***	6.66	253.75***	16.34	214.65***	17.33	125.30***	6.39	93.75***	7.81
Controls	Yes		Yes		Yes		Yes		Yes		Yes	
*R*^2^	0.13		0.03		0.18		0.17		0.15		0.16	
Number of observations	4226		4226		4226		4226		4226		4226	
*Panel D: age 10–12*												
Separation	66.01***	6.29	− 3.66	4.24	− 72.44***	5.42	− 3.92	7.92	− 7.27	5.26	14.97*	6.66
Intercept	90.76***	7.38	37.66	4.86	75.07***	6.43	176.76***	9.36	92.05***	6.46	158.16***	7.77
Controls	Yes		Yes		Yes		Yes		Yes		Yes	
*R*^2^	0.03		0.02		0.06		0.05		0.08		0.07	
Number of observations	4705		4705		4705		4705		4705		4705	
*Panel E: age 12–14*												
Separation	64.24***	5.97	− 10.54**	4.10	− 73.93***	5.30	11.99^+^	8.85	− 12.04*	5.01	16.80*	7.20
Intercept	85.04***	7.96	38.11***	5.47	77.01***	7.06	197.54***	11.79	119.71***	6.81	183.64***	9.60
Controls	Yes		Yes		Yes		Yes		Yes		Yes	
*R*^2^	0.17		0.03		0.06		0.10		0.06		0.08	
Number of observations	4813		4813		4813		4813		4813		4813	

Longitudinal study of Australian Children. *K* Cohort. Panel A includes waves 1–2; Panel B, waves 2–3; Panel C, waves 3–4; Panel D, waves 4–5 and Panel E, waves 5–6

Models control for the lagged dependent variable, child sex, child age, maternal education, paternal education, mother’s class, father’s class, language spoken at home, number of siblings, region of residence, and residential parent re-partnering

^+^*p* < 0.10 *p < 0.05, **p < 0.01, ***p < 0.001

### Additional Analyses II: Time Use During Weekends

We estimated additional analyses for weekends, using data for the three waves containing weekend diary measures, namely wave 1 (at age 4), wave 2 (at age 6) and wave 3 (at age 8). Data for the last three waves of our study (ages 10, 12, and 14) were not analyzed on weekends because there were very few weekend observations for these recent waves, which restricted a robust estimation of parental separation effects on time-use measures.

**Fig. 6:**
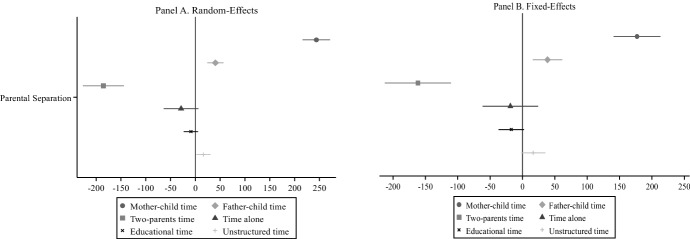
Regression models of children’s time with parents, alone, and in activity types on weekends (waves 1–3). *Source* Longitudinal Study of Australian Children. *K* Cohort, waves 1–3, weekend time-diaries. *Note:* Models control for child gender, child age, maternal education, paternal education, mother’s class, father’s class, language spoken at home, number of siblings, region of residence, and residential parent re-partnering/remarriage. CI included at the 95% level (number of observations = 7738)

**Fig. 7:**
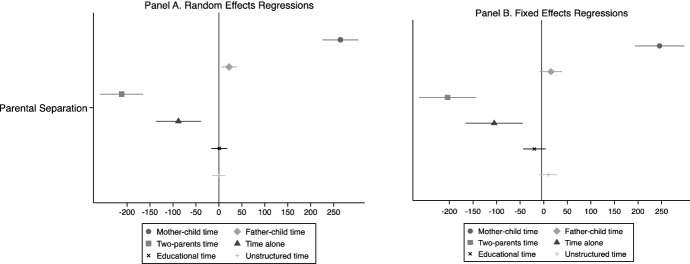
Regression models of children’s time with parents, alone, and in activity types on weekdays (waves 1–3). *Source* Longitudinal Study of Australian Children. *K* Cohort, waves 1–3, weekday time-diaries. *Note:* Models control for child gender, child age, maternal education, paternal education, mother’s class, father’s class, language spoken at home, number of siblings, region of residence, and residential parent re-partnering/remarriage. CI included at the 95% level (number of observations = 7738)

Figure 6 presents analyses on the effect of parental separation on time use on weekends for waves 1–3. For comparison, Fig. 7 presents the same analyses for weekdays for waves 1–3. With some difference in magnitude and statistical significance, the results of random effects (Panel A) and fixed effects (Panel B) are quite consistent. On weekends, transitioning to separation leads father–child solo time to an increase of about 45 min per day after separation (*p* < 0.001) and mother–child solo time to increases of more than 200 min in the random effects models and less than 200 min in the fixed effects models (*p* < 0.001). These results show that, after separation, the gap between father– and mother–child time is smaller on weekends, compared to the gap after separation on weekdays. However, these results need to be put in context. As previous research has shown, fathers are disproportionately active in childcare activities on weekends (Hook & Wolfe, 2012). Additional analyses (not shown) reveal that, both in non-separated and separated families, differences in time with children between fathers and mothers are smaller on weekends than on weekdays. In terms of activity types, we see generally consistent patterns between weekdays and weekends once unobserved heterogeneity is accounted for. Overall, additional analyses on weekends show some differences with weekdays regarding mother– and father–child time, but on weekends we still see a clear gendered pattern in parental time use after separation.

### Robustness Checks

We carried out three final robustness checks, not shown in the study. First, we replicated our models by including control variables indexing mothers’ and fathers’ paid work hours. We decided not to include employment status in the main models because they fall into the causal pathway between parental separation and time investments: time devoted to paid work should affect time with children as well as be affected by separation. Therefore, the inclusion of time in paid work could potentially downward our estimates, particularly those of parental time.

Second, we analyzed whether the resident parent’s re-partnering alters the effects of separation on time use. One fourth of the couples who separate are observed to find a new partner within the period of observation. This might potentially alter our parent–child time estimates and might be causing the increase in two-parent time over the years since divorce. We speculate two reasons for this increase of two-parent time. First, perhaps once tensions associated with marital dissolution fade away, biological parents are willing to spend more time together with their children, as the short-term negative effects of divorce over families’ mental health, stress, and conflict vanish after 1 or 2 years (e.g., Leopold & Kalmijn, 2016). And second, with years since separation, children might consider the stepfather as the father. We cannot fully answer this question with our data. Future studies should further examine differences in parent–child time among non-residential biological parents, social parents and stepparents.

Finally, we replicated our analyses separately for waves 1–3 and waves 4–6. We did this final sensitivity test because time-diaries had a change in reporter between waves three and four, being the mother filling out the diary in the first three waves, and the child in subsequent waves. None of these analyses changed the conclusions of this study.

## Discussion

This study is to our knowledge the first long-term longitudinal examination of the effects of parental separation on parental involvement and children’s time use. Previous studies focused on the effect of separation on parent–child *contact* or parental *involvement*. Instead, we present a new life-course approach using unique time-diary longitudinal data and focusing on time use among both parents and children. Our research design unlike in previous related studies allows us to control for omitted-variable bias, which has been an essential concern within the divorce literature (McLanahan et al., 2013). This study has four main findings.

First, we find a clear *gendered shift* in the composition of parental time after separation: divorce leads to a huge increase in mother–child time, paralleled by a similar decrease in two-parent time. Father–child time remains low and virtually unaffected by parental separation during weekdays. These results support our Hypothesis 1, except for father–child time. That father–child time does not change with separation is somehow surprising and against our theoretical predictions and previous studies (Cheadle et al., 2010; Gratz, 2017; Kalmijn, 2015). Our longitudinal approach complements the cross-sectional study of Kalil et al. (2014), which shows that single mothers do more solo childcare than partnered mothers in the US. Consistent with a “time poverty” perspective (see Pepin et al., 2018; Chatzitheochari & Arber, 2012), we find a sharp increase of gender inequalities in childcare after separation. These inequalities are important in explaining women’s disadvantages in their income, leisure, and paid work time.

Second, we find that parental separation leads to a substantial change in children’s time across activity types. Our results show that, after divorce, children’s time in *unstructured leisure* (i.e., TV, smartphone usage, unstructured play) increases and their time in *educational activities* (i.e., reading, educational games or structured sports) declines. These results are in line with Hypothesis 2. Previous studies found that educational activities are the most relevant inputs for the child cognitive development (Cano et al., 2019; Fiorini & Keane, 2014). On the contrary, child time in certain forms of unstructured leisure has been found to lead to sleep disruptions, socio-emotional problems, and health disadvantages (Beyens et al., 2018; Kelly et al., 2019). The effect of separation on children’s time use could mediate these negative effects of parental separation on child development. Therefore, focusing not only on parental time investments, but also on “what children do” makes a novel contribution to the divorce literature on child well-being (e.g., Harkonen et al., 2017; Kim, 2011). Future studies on divorce and child well-being should take these results into consideration.

Third, our analyses reveal that the effects of parental separation on time use differ between boys and girls. After separation, boys lose more time with parents and in educational activities than girls and increase time in unstructured leisure more than girls. These results generally support Hypothesis 3b and suggest that boys are more negatively affected by separation than girls. Previous studies examined several mechanisms that might explain why boys perform worse than girls at school, including teacher bias (Legewie & DiPrete, 2012), gendered behavioral outcomes (DiPrete & Jennings, 2012), the role of gender egalitarianism and culture (Guiso et al., 2008) or differential parental investments (Baker & Milligan, 2016). Our study contributes to research on the underachievement of boys in and outside school (Chetty et al., 2020) by showing the heterogenous impact of separation on boys’ and girls’ time use. Why boys are more vulnerable than girls to separation is an important area of study for future research.

Fourth, we find that the effect of parental separation on parents’ and children’s time use is temporary, with strong effects in the short run, and with a return to pre-separation levels after 2–4 years, particularly regarding mother–child time. Children’s time use follows a different life-course pattern. The negative effects of separation on children’s developmental time use are observed already before separation and stabilize afterwards, when children are no longer living with the two parents, but with one (primarily the mother). That children increase their time in unstructured activities mainly before separation should reflect parents’ pre-separation conflict, tension, and stress. This is in line with the “selection perspective” (Amato, 2000), suggesting that pre-separation declines in children’s time in developmental activities reflect socioemotional or behavioral problems (e.g., depression, hyperactivity) that are observed right before divorce. The fact that children’s time use stabilizes after divorce can also suggest that the effects of divorce are not due to selection, but to stress linked to divorce, as in the “divorce-stress-adjustment perspective” (Amato, 2000).

Finally, additional analyses on weekends show that the gap between father– and mother–child time after separation is smaller than the same gender gap after separation during weekdays. Yet, these results need to be contextualized. Father–child time is higher on weekends than on weekdays, not only in single-parent families, but also in two-parent families (Hook & Wolfe, 2012). Additionally, we did not find clear changes in child time use across activity types after separation when comparing weekdays to weekends. Future research should further investigate the role of separation in parents’ and children’s time use during weekends.

Our study has some caveats that we need to mention. First, the LSAC time-diaries were completed by parents in waves 1–3, using paper-based time-diaries, while in waves 4–6 children filled the diaries electronically via digital tablets, without a pre-coded list of activities. To overcome potential bias in this regard, we excluded activities that are not meaningful to compare over the life course (e.g., breastfeeding, changing nappies), focusing on harmonized measures over time (Mullan, 2014). We conducted supplementary analyses creating two different subsamples depending on the type of time-diary collection, which yielded similar results to those reported in our main analyses. It is important to highlight that all children (in both single and two-parent families) filled the same types of diaries. Second, the LSAC time-diaries did not allow us to clearly distinguish between biological and stepparents when examining ‘with who’ children spent time. To account for this, we controlled for re-partnering in our analyses, and conducted robustness checks with a subsample of children who did not experience re-partnering. These analyses provided equivalent results to our main analyses. Third, although having a long period of data collection throughout 10 years of bi-annual observations is a clear strength of our paper, it also imposes some limitations to our empirical estimates. A key issue here is that children’s change physically and mentally from age 4 to 14, hereby affecting the fixed effect’s assumption of constant unobserved heterogeneity. For this reason, we have complemented our main analyses with another set of models that do not hold such assumption (i.e., value-added models) and have discussed these results in our study. We hope future research will be able to capture differences in children’s time with different parental figures (i.e., social and biological), adding to a relevant emerging field in the family literature (e.g., Kalmijn et al., 2019).

Taken all together, our study provides novel evidence on how parental separation impacts family life and parents’ and children’s daily activities. These findings have strong social policy implications. First, separation not only leads mothers to experiencing a motherhood wage penalty, but also a *time penalty*. Promoting gender equality in caring responsibilities after divorce (e.g., via shared child joint custody) could bring improvements in mother’s career advancements, with separated fathers potentially working more on caring for children. Second, the fact that boys’ school-relevant activities (i.e., educational versus unstructured leisure) are disproportionally harmed by separation is relevant to inform policy makers and educators regarding young people’s gendered educational and behavioral outcomes. We hope our study will inspire new research on the (gendered) nature of divorce in shaping parents’ and children’s lifestyles and well-being over the life course.

## Acknowledgements

We gratefully acknowledge funding support from the projects CSO2016-80484-R (funded by the Spanish Ministry of Science; PIs: Pau Baizán and Clara Cortina) and ‘Social Inequalities in Girls’ and Boys’ Daily Activities and Skills Accumulation’ (funded by Trinity College Dublin: PI: Pablo Gracia). This paper uses data from the Longitudinal Study of Australian Children, a survey conducted in partnership between the Department of Social Services, the Australian Institute of Family Studies and the Australian Bureau of Statistics. Previous versions of the study were presented at Trinity College Dublin (2019), the Economic and Social Research Institute (2019), Goethe University of Frankfurt (2019), University of Rotterdam (2019), European Consortium for Sociological Research Conference (2019), Netherlands Interdisciplinary Demographic Institute (2020), University of Oxford (2020), University of St. Andrews (2021), Population Association of America Conference (2021) and International Association for Time Use Research Conference (2021). We thank participants at these events, and particularly Martin O’Flaherty, Christian Czymara and Lena Scherf, for their valuable comments and assistance on our study. Finally, we thank editors and two anonymous reviewers from the *European Journal of Population* for their excellent comments and suggestions on earlier versions of our paper.

## Appendix

See Tables 3, 4, 5, 6 and Figs. 7.

**Table 3:** Correspondence between activities and time-use categories

Category	Activities details	Location
Mother–child solo time	Any activity with the mother and without the father	Any place
Father–child solo time	Any activity with the father and without the mother	Any place
Two-parents’ time	Any activity in presence of both the mother and the father	Any place
Time alone	Any activity alone, without the presence of others	Any place
Educational activities	Reading, study, homework (electronic or not), going to theatre, opera, concerts, cinema, library time, doing music, dance, theatre, artistic activities, structured sports, language lessons	Outside School
Unstructured leisure	Unstructured playing, watching TV, playing video games, video watching, using mobile phones, illegal activities, browsing on the internet (non-schooling related), social media activities	Outside School

Longitudinal study of Australian Children. *K* Cohort, waves 1–6 (2004–2014)

**Table 4:** Random effect models. Children’s daily minutes. Parental separation average and long-term effects

	Mother–child solo time		Father–child solo time		Two-parents’ time		Time alone		Educational activities		Unstructured activities	
	*β*	SE	*β*	SE	*β*	SE	*β*	SE	*β*	SE	*β*	SE
Separation average effects	106.94***	7.87	− 5.47	3.73	− 96.16***	9.13	− 14.05	10.32	− 7.69^+^	4	17.35***	4.63
Girl	5.23	3.76	− 9.37***	1.71	1.76	4.43	− 12.98**	4.93	8.78***	1.79	− 31.45***	2.17
6 years old	− 90.16***	4.78	− 11.32***	2.32	− 35.25***	5.5	21.89***	6.27	− 55.00***	2.54	− 102.98***	2.85
8 years old	− 89.37***	4.98	− 12.07***	2.41	6.4	5.72	24.57***	6.52	− 51.90***	2.64	− 85.09***	2.96
10 years old	− 192.32***	4.89	− 22.76***	2.37	− 180.22***	5.62	− 109.47***	6.4	− 110.61***	2.58	− 26.89***	2.91
12 years old	− 207.64***	7.15	− 23.89***	3.44	− 166.81***	8.25	− 27.84**	9.37	− 57.14***	3.73	− 15.51***	4.24
14 years old	− 216.64***	7.36	− 28.59***	3.54	− 167.83***	8.5	45.64***	9.65	− 90.18***	3.84	15.79***	4.36
Mother’s college	0.37	4.31	3.42^+^	1.99	− 8.35^+^	5.06	12.23*	5.65	18.17***	2.08	− 12.67***	2.5
Father’s college	1.87	4.42	0.49	2.03	− 9.06^+^	5.19	1.22	5.79	13.97***	2.13	− 10.22***	2.56
Mother: manager or professional occupation	− 12.64*	6.08	8.11**	2.91	− 10.3	7.02	31.38***	7.97	12.56***	3.14	− 6.24^+^	3.59
Mother: intermediate class	− 3.7	6.23	− 0.31	2.98	4.86	7.2	33.62***	8.17	8.26*	3.22	− 7.37*	3.68
Mother: not in employment	33.78***	5.42	− 13.37***	2.58	− 12.32*	6.28	29.66***	7.1	13.31***	2.77	3.36	3.19
Father: manager or professional occupation	2.38	5.34	− 5.46*	2.55	6.74	6.19	33.43***	7	6.58*	2.74	− 0.95	3.15
Father: intermediate class	2.14	5.86	− 7.02*	2.79	11.55^+^	6.79	14.76^+^	7.68	3.66	3	1.51	3.45
Father: not in employment	− 66.50***	8.09	21.45***	3.85	43.91***	9.37	9.4	10.6	3.64	4.13	20.08***	4.77
Resident parent re-partners	− 99.92***	15.75	19.11*	7.52	82.62***	18.24	41.46*	20.65	18.36*	8.1	− 12.43	9.3
Intercept	288.71***	9.54	70.50***	4.47	254.77***	11.13	183.69***	12.51	163.11***	4.76	197.45***	5.58
*Within R*^*2*^	0.18		0.03		0.15		0.07		0.16		0.17	
*Number of observations*	14,862		14,862		14,862		14,862		14,862		14,862	
− 10/− 8 Years before separation (ref)												
− 6/− 4 Years before separation	16.38*	7.77	5.49	3.66	− 3.84	9.05	− 21.89*	10.19	− 2.23	3.91	0.33	4.56
− 2 Years before separation	− 3.93	10.32	7.71	4.93	− 6.45	11.96	0.1	13.54	− 4.49	5.33	7	6.1
0 Years since separation	134.65***	10.2	4.19	4.86	− 116.47***	11.82	− 35.20**	13.37	− 6.71	5.25	20.02***	6.02
+ 2 Years after separation	104.55***	12.42	− 7.35	5.94	− 83.18***	14.38	6.56	16.29	− 4.5	6.42	16.80*	7.34
+4/+6 Years after separation	48.90***	12.51	− 16.54**	5.93	− 63.73***	14.52	3.36	16.4	− 10.16	6.38	20.01**	7.36
Girl	5.04	3.76	− 9.41***	1.71	1.87	4.43	− 12.89**	4.93	8.81***	1.78	− 31.56***	2.17
6 years old	− 90.66***	4.78	− 11.51***	2.32	− 34.84***	5.5	22.23***	6.27	− 55.03***	2.54	− 103.06***	2.85
8 years old	− 89.85***	4.98	− 12.26***	2.42	6.98	5.74	24.14***	6.54	− 52.03***	2.64	− 85.26***	2.97
10 years old	− 191.83***	4.9	− 22.61***	2.38	− 180.08***	5.65	− 110.82***	6.43	− 110.82***	2.6	− 27.17***	2.92
12 years old	− 202.56***	7.23	− 22.36***	3.47	− 169.31***	8.35	− 32.26***	9.48	− 57.24***	3.77	− 15.50***	4.28
14 years old	− 210.05***	7.47	− 26.51***	3.59	− 171.02***	8.63	41.17***	9.8	− 90.20***	3.9	15.81***	4.43
Mother’s college	0.31	4.31	3.58^+^	1.98	− 8.27	5.06	11.92*	5.66	18.12***	2.08	− 12.61***	2.5
Father’s college	1.76	4.42	0.49	2.03	− 8.97^+^	5.19	1.15	5.8	13.89***	2.13	− 10.14***	2.56
Mother: manager or professional occupation	− 12.06*	6.07	8.27**	2.91	− 10.52	7.03	30.84***	7.97	12.45***	3.15	− 6.20^+^	3.59
Mother: intermediate class	− 3.03	6.23	− 0.11	2.98	4.46	7.2	33.00***	8.17	8.21*	3.22	− 7.33*	3.68
Mother: not in employment	33.98***	5.41	− 13.28***	2.58	− 12.43*	6.27	29.42***	7.1	13.26***	2.77	3.29	3.19
Father: manager or professional occupation	5.52	5.37	− 4.45^+^	2.56	4.9	6.22	31.75***	7.04	6.86*	2.76	− 0.69	3.17
Father: intermediate class	4.59	5.87	− 6.09*	2.8	10.08	6.81	13.41^+^	7.7	3.88	3.01	1.79	3.47
Father: not in employment	− 66.12***	8.08	21.45***	3.84	43.67***	9.37	9.58	10.6	4.08	4.14	20.03***	4.77
Resident parent re-partners	− 78.04***	16.01	23.69**	7.64	67.39***	18.55	29.06	21	17.76*	8.22	− 13.04	9.45
Intercept	285.02***	9.59	69.07***	4.49	256.45***	11.19	187.06***	12.58	163.22***	4.78	197.16***	5.61
*Within R*^*2*^	0.18		0.03		0.15		0.07		0.16		0.17	
*Number of observations*	14,862		14,862		14,862		14,862		14,862		14,862	

Longitudinal study of Australian Children. *K* Cohort, waves 1–6, weekdays time-diaries

^+^*p* < 0.10* *p* < 0.05,* **p* < 0.01,* ***p* < 0.001

Results from 12 separated random effects GLS models. Six models estimate separation average effects from intact to a separated family (above) and six additional models estimate the long-term separation effects including the years before and after separation (below) to predict children’s daily minutes. In all 12 models, the reference category of *child age* is “4 years old” and the reference category of both *mother’s class* and *father’s class* is “working-class occupation”. All models control additionally for language spoken at home and the country’s region of residence

**Table 5:** Fixed effect models. Children’s daily minutes. Parental separation average and long-term effects

	Mother–child solo time		Father–child solo time		Two− parents’ time		Time alone		Educational activities		Unstructured activities	
	*β*	SE	*β*	SE	*β*	SE	*β*	SE	*β*	SE	*β*	SE
Separation average effects	90.78***	10.28	− 6.48	4.95	− 93.36***	11.71	− 3.31	13.24	-8.61	5.4	21.80***	6.07
6 years old	− 91.15***	5.07	− 9.58***	2.44	− 34.56***	5.78	19.08**	6.53	− 56.64***	2.66	− 102.74***	3
8 years old	− 90.07***	5.32	− 10.57***	2.56	5.44	6.06	20.87**	6.85	− 54.77***	2.79	− 86.11***	3.14
10 Years old	− 192.09***	5.32	− 20.20***	2.56	− 179.03***	6.06	− 113.58***	6.85	− 112.34***	2.8	− 29.64***	3.14
12 years old	− 207.66***	8.41	− 24.24***	4.05	− 166.35***	9.58	− 30.83**	10.83	− 58.31***	4.42	− 14.20**	4.97
14 years old	− 217.34***	8.64	− 29.64***	4.16	− 165.70***	9.84	39.22***	11.12	− 93.43***	4.53	18.72***	5.1
Mother’s college	− 10.79	13.07	2.27	6.3	20	14.89	14.71	16.83	− 1.32	6.86	6.39	7.72
Father’s college	19.95	15.72	− 8.51	7.57	− 10.15	17.9	15.88	20.23	7.56	8.25	10.25	9.28
Mother: manager or professional occupation	− 7.33	7.24	2.04	3.49	− 18.45*	8.25	33.73***	9.32	15.57***	3.8	− 3.54	4.28
Mother: intermediate class	− 4.34	7.42	− 3.63	3.57	4.85	8.45	37.68***	9.55	10.68**	3.9	− 4.29	4.38
Mother: not in employment	27.85***	6.97	− 10.76**	3.36	− 17.61*	7.94	34.40***	8.97	11.15**	3.66	2.18	4.11
Father: manager or professional occupation	− 1.74	6.7	− 5.63^+^	3.23	12.02	7.63	30.56***	8.63	0.81	3.52	6.31	3.96
Father: intermediate class	− 0.38	7.44	− 6.67^+^	3.58	14.87^+^	8.48	20.49*	9.58	4.41	3.91	5.04	4.39
Father: not in employment	− 48.96***	10.68	17.23***	5.15	36.27**	12.17	25.05^+^	13.75	5.37	5.61	15.06*	6.31
Resident parent re-partners	− 82.18***	19.21	2.72	9.25	82.24***	21.88	62.04*	24.73	21.07*	10.08	− 17.71	11.34
Intercept	333.72***	31.42	60.30***	15.13	225.36***	35.79	140.59***	40.44	201.69***	16.49	153.44***	18.55
*Within R*^2^	0.19		0.02		0.18		0.06		0.17		0.18	
*Number of observations*	2054		2054		2054		2054		2054		2054	
− 10/ −8 Years before separation (ref)												
− 6/− 4 Years before separation	− 23.86	25.01	14.44	12.06	− 45.87	28.53	− 10.89	32.25	− 17.14	13.16	9.97	14.8
− 2 Years before separation	− 59.18*	26.81	16.02	12.93	− 41.5	30.58	16.13	34.56	− 21.03	14.1	18.26	15.86
0 Years since separation	78.07**	26.93	15.93	12.99	− 152.05***	30.72	− 16.72	34.72	− 24.19+	14.16	36.42*	15.93
+2 Years after separation	40.27	28.75	3.76	13.87	− 122.07***	32.8	26.33	37.07	− 22.62	15.12	34.53*	17.01
+ 4/+ 6 years after separation	− 29.44	29.46	− 10.93	14.21	− 89.69**	33.61	20.44	37.98	− 33.42*	15.49	38.78*	17.43
6 years old	− 90.46***	5.11	− 10.12***	2.46	− 33.22***	5.83	19.30**	6.58	− 56.20***	2.69	− 103.14***	3.02
8 years old	− 89.24***	5.36	− 11.27***	2.59	7.01	6.12	20.38**	6.92	− 54.37***	2.82	− 86.71***	3.17
10 years old	− 189.40***	5.38	− 20.51***	2.59	− 178.32***	6.14	− 114.99***	6.94	− 111.78***	2.83	− 30.47***	3.18
12 years old	− 197.18***	8.56	− 22.57***	4.13	− 169.80***	9.76	− 35.71**	11.03	− 57.00***	4.5	− 15.32**	5.06
14 years old	− 205.01***	8.82	− 27.39***	4.25	− 170.12***	10.06	34.57**	11.37	− 91.81***	4.64	17.51***	5.22
Mother’s college	− 9.8	13.05	2.44	6.29	19.51	14.89	14.19	16.83	− 1.25	6.86	6.37	7.72
Father’s college	19.55	15.69	− 8.8	7.57	− 9.52	17.9	16.2	20.23	7.63	8.25	10.18	9.28
Mother: manager or professional occupation	− 7.04	7.23	2.17	3.49	− 19.07*	8.25	33.38***	9.32	15.41***	3.8	− 3.52	4.28
Mother: intermediate class	− 3.81	7.41	− 3.34	3.57	4.13	8.45	37.04***	9.55	10.58**	3.9	− 4.25	4.38
Mother: not in employment	28.44***	6.95	− 10.65**	3.35	− 17.89*	7.93	34.18***	8.97	11.22**	3.66	2.09	4.11
Father: manager or professional occupation	5.56	6.78	− 3.91	3.27	8.74	7.74	28.03**	8.75	1.83	3.57	6.05	4.01
Father: intermediate class	5.53	7.48	− 5.06	3.61	11.98	8.54	18.37^+^	9.65	5.27	3.94	4.87	4.43
Father: not in employment	− 46.59***	10.67	17.74***	5.14	35.79**	12.17	24.23^+^	13.75	6	5.61	14.95*	6.31
Resident parent re-partners	− 50.95**	19.61	11.14	9.46	60.80**	22.38	46.89^+^	25.29	21.29*	10.32	− 18.55	11.6
Intercept	331.88***	31.54	57.12***	15.21	234.32***	35.98	144.41***	40.67	203.48***	16.59	152.16***	18.66
*Within R*^2^	0.2		0.02		0.18		0.06		0.17		0.18	
*Number of observations*	2054		2054		2054		2054		2054		2054	

Longitudinal Study of Australian Children. K Cohort, waves 1–6 (2004–2014)

^+^*p* < 0.10 **p* < 0.05, ***p* < 0.01, ****p* < 0.001

Results from 12 separated fixed effects GLS models. Six models estimate separation average effects from intact to a separated family (above) and six additional models estimate the long-term separation effects including the years before and after separation (below) to predict children’s daily minutes. In all 12 models, the reference category of *child age* is “4 years old” and the reference category of both *mother’s class* and *father’s class* is “working-class occupation”. All models control additionally for language spoken at home and the country’s region of residence

**Table 6:** Random effects regression models of child time use, interactions with child gender

	Mother–child solo time		Father–child solo time		Two-parents’ time		Time alone		Educational activities		Unstructured activities	
	*β*	SE	*β*	SE	*β*	SE	*β*	SE	*β*	SE	*β*	SE
*Panel A: average effects*												
Boys												
Separation	105.72***	11.65	− 9.28	5.72	− 103.53***	13.37	− 2.13	15.16	− 9.78^+^	5.73	24.05***	7.07
Intercept	296.68***	13.55	66.79***	6.53	244.02***	15.62	168.98***	17.61	154.85***	6.49	197.90***	8.09
Controls	Yes		Yes		Yes		Yes		Yes		Yes	
Within *R*^2^	0.2		0.01		0.17		0.07		0.18		0.2	
Number of observations	7476		7476		7476		7476		7476		7476	
Girls												
Separation	105.36***	10.6	− 1.92	4.83	− 90.54***	12.49	− 24.14^+^	14.05	− 6.01	5.57	11.75*	5.98
Intercept	284.66***	12.9	64.76***	5.87	268.96***	15.32	186.00***	17.16	180.13***	6.75	166.28***	7.34
Controls	Yes		Yes		Yes		Yes		Yes		Yes	
Within *R*^2^	0.19		0.02		0.19		0.06		0.17		0.15	
Number of observations	7386		7386		7386		7386		7386		7386	
*Panel B: long-term effects*												
Years before/after separation (Ref.: − 10/− 8)												
1.5	31.75**	11.14	− 1.01	5.22	− 10.05	12.83	− 43.55**	14.46	− 5	5.54	− 3.77	6.47
−2	11	15.25	1.02	7.27	33.87^+^	17.55	− 12.36	19.88	− 9.09	7.83	2.8	8.96
0	142.18***	14.24	− 1.67	6.77	− 125.41***	16.39	− 33.11^+^	18.56	− 11.55	7.29	26.39**	8.35
2	104.61***	17.29	− 0.76	8.25	− 78.97***	19.89	− 13.23	22.54	− 10.68	8.89	36.85***	10.16
0.6667	60.22***	17.64	− 21.35*	8.36	− 59.34**	20.31	− 13.39	22.97	− 11.47	8.95	24.75*	10.32
Girls	7.35^+^	4.11	− 10.39***	1.87	3.05	4.75	− 15.59**	5.3	8.06***	1.92	− 31.22***	2.34
Years before/after separation X girls												
1.5	− 33.25*	15.42	13.85^+^	7.23	15.42	17.76	36.96^+^	20.02	4.18	7.68	10.42	8.96
-2	− 33.61	20.77	14.59	9.9	− 71.25**	23.9	20.28	27.08	7.87	10.66	10.19	12.2
0	− 11.31	19.51	13.23	9.28	6	22.45	− 28.87	25.42	3.82	9.98	− 12.64	11.44
2	1.7	23.44	− 11.93	11.19	− 14.49	26.97	24.84	30.57	8.32	12.06	− 41.07**	13.78
0.6667	− 14.99	22.87	7.39	10.83	− 15.99	26.33	27.26	29.77	1.33	11.59	− 10.49	13.38
Intercept	293.03***	7.76	63.75***	3.58	258.69***	8.95	234.45***	10.04	178.80***	3.74	198.22***	4.46
Controls	Yes		Yes		Yes		Yes		Yes		Yes	
*Within R*^2^	0.17		0.02		0.15		0.07		0.15		0.17	
*Number of observations*	14,862		14,862		14,862		14,862		14,862		14,862	

Longitudinal Study of Australian Children. *K* Cohort, waves 1–6 (2002–2014). ^+^*p* < 0.10 **p* < 0.05, ***p* < 0.01, ****p* < 0.001

Panel A shows regression estimated separately in two subsamples of girls and boys (i.e., 12 regressions, six per sex). Panel B shows six regressions where each of them includes an interaction term between child’s sex and separation in a categorical form that indexes years before and after. All models control for child sex, child age, maternal education, paternal education, mother’s class, father’s class, language spoken at home, number of siblings, region of residence, and residential parent re-partnering. Confidence intervals are included at the 95% level (number of observations = 14,862)

6 and

## Footnotes

1 Throughout the paper we use divorce, separation, or union dissolution as interchangeable synonyms.

2 An example of the LSAC time diaries in waves 1–3 can be seen here: http://data.growingupinaustralia.gov.au/studyqns/wave1qns/TUD14.pdf. For waves 3–6, see: https://growingupinaustralia.gov.au/sites/default/files/tp13.pdf pp. 21–45).

3 Diaries did not distinguish between copresence with social or biological father and social or biological mother; only 1.3% of the observations in the total sample (9.2% in the separation sample) lived with a step-parent (normally, a step-father).

4 We multiplied the resulting number by 1 in each of the slots marked with our activities of interest. In waves 1–3, we calculated time by multiplying each of the 96 diary slots marked with one of our activities of interest by 15. In waves 4–6, where diaries were not divided into pre-defined number of slots, we first used the difference between activities’ end-times and activities’ start-times to calculate time duration within the defined sequences of starting activity hours.

5 As in previous research (Cano et al., 2019), when multiple activities were carried on at the same time, we divided the number of activities by the specified time. For example, if the child reported to be doing homework and watching TV between 4 and 5 pm, we counted 30 min to educational activities and 30 min to unstructured leisure.

6 We further conducted lagged-inputs plus value-added models for our five child-age subsamples (for details, see Todd and Wolpin, 2007). These results were consistent with the analyses presented in Table 2.
